# Pregnant Women’s Experiences and Perceptions of the Impact of Exercise on Mental Health During Pregnancy—A Qualitative Study

**DOI:** 10.3390/healthcare14050678

**Published:** 2026-03-07

**Authors:** Manuela Filipec, Marko Bodrožić, Sania Almousa

**Affiliations:** 1 Department of Physiotherapy, University North, 42000 Varaždin, Croatia; 2 Department of Physical Medicine and Rehabilitation, Clinical Hospital Sveti Duh, 10000 Zagreb, Croatia; 3 School of Physiotherapy, RCSI University of Medicine and Health Sciences, D02 YN77 Dublin, Ireland

**Keywords:** exercise, mental health, pregnancy

## Abstract

**Highlights:**

**What are the main findings?**

- Pregnant women perceived exercise as a key self-care strategy that supported emotional regulation, reduced stress and anxiety, and enhanced overall psychological well-being during pregnancy.

**What are the implications of the main findings?**

- Maternity care providers should frame exercise not only as a physical health intervention but also as a meaningful tool for supporting maternal mental health during pregnancy.

**Abstract:**

**Background:** Exercise during pregnancy is known to benefit physical and mental health. However, pregnant women’s lived experiences of its psychological impact remain insufficiently explored. This study aimed to explore pregnant women’s experiences and perceptions of how exercise influences mental health during pregnancy. **Methods:** A qualitative study design was employed. Pregnant women were recruited using purposive sampling from a clinical hospital setting between March and September 2025. Eligible participants met predefined inclusion and exclusion criteria. Data saturation guided sample size (N = 38). Data were collected through semi-structured online interviews. Interviews were audio-recorded, transcribed verbatim, and analysed using inductive thematic analysis. **Results:** Four major participant-derived themes emerged: emotional regulation and mood stabilization, reduction of anxiety and depressive symptoms, enhanced self-confidence and body acceptance, and increased self-efficacy and sense of control. These themes illustrate the range of psychological benefits associated with maintaining exercise during pregnancy. **Conclusions:** This study highlights the psychological meanings pregnant women attribute to exercise, extending beyond its established physical benefits. These insights underscore the importance of integrating mental health perspective into prenatal physical activity counselling and support the development of more individualized, patient-centered prenatal care strategies.

## 1. Introduction

Pregnancy represents a period of profound physical, psychological, and social change [1,2]. While it is often associated with joy and anticipation, it also brings increased vulnerability to mental health difficulties. Although it is commonly associated with happiness and expectation, it is also a time of heightened sensitivity to mental health challenges [3]. The World Health Organization (WHO) defines maternal mental health as a state of well-being in which a woman recognizes her abilities, is able to manage the normal stresses of life, work productively, and contribute to her community [4]. Maternal mental health disorders are widely acknowledged as a major global public health concern, with a concerning rise in prevalence during the antenatal period. According to the WHO, approximately 10% of pregnant women worldwide experience a mental health disorder, with rates increasing to around 15.6% in developing countries during pregnancy [5]. Among the most prevalent mental health conditions during pregnancy are stress, anxiety, and depression. Evidence from the literature indicates that the prevalence of antenatal depression ranges from 6% to 25% [6], while anxiety during pregnancy has been reported to affect between 8.5% and 15.2% of women [7,8,9]. An increasing body of research highlights exercise as an effective, accessible, and non-pharmacological approach to supporting and improving mental health during pregnancy. Regular exercise has been associated with reductions in symptoms of depression, anxiety, stress, and pregnancy-related fatigue, alongside improvements in mood, sleep quality, cognitive functioning, and overall well-being [10]. Beyond physiological effects, exercise promotes the release of endorphins, enhances blood circulation, and supports metabolic regulation [10]. Also, psychologically it strengthens self-efficacy, perceived control, and positive body image, while socially, participation in group-based prenatal exercise programs can provide emotional support and foster social connections [10,11,12]. Barba-Müller et al. (2019) report that pregnancy is accompanied by significant adaptations in the maternal brain, including changes in neuroplasticity that influence caregiving behaviors, emotional regulation, and social cognition [13,14]. While these neurological adaptations are considered a normal aspect of pregnancy, they may also increase susceptibility to mental health difficulties. Exercise has been shown to activate several neurobiological mechanisms that promote neuroplasticity including increased production of neurotrophic factors such as brain-derived neurotrophic factor (BDNF), enlargement of hippocampal and cortical volumes, reductions in neuroinflammation, and enhanced connectivity within neural pathways involving dopamine, norepinephrine, and serotonin [13,15]. Through these mechanisms, exercise may influence the underlying pathophysiology of depression and anxiety, positioning it as a promising non-pharmacological intervention for reducing the risk and severity of mental health symptoms during pregnancy [13,15]. In addition to neurobiological effects, psychosocial mechanisms may also contribute to the mental health benefits of exercise. These include enhanced self-efficacy, increased self-confidence, feelings of accomplishment, improved self-esteem, a more positive body image, and overall improvements in physical quality of life [13,15].

Despite its well-established benefits physical activity, typically declines as pregnancy progress. This decline is multifactorial and can be attributed to a combination of physiological, psychological, cultural, and social influences. Health care advice (insufficient advice/information), personal attitudes (safety/fear of harming the fetus), cultural beliefs, family pressures, and social norms have all been identified as important determinants that may either promote or inhibit participation in exercise during pregnancy [16]. Moreover, the influence of advice received from healthcare providers is particularly significant, with studies showing that women who are guided about safe exercise during pregnancy are more likely to remain active, whereas those advised to stop or slow down activity tend to reduce or discontinue exercise altogether [16]. Thus, engagement in physical activity during pregnancy is not shaped solely by biomedical recommendations. Sociocultural factors—including cultural beliefs about safety, protection, and risk avoidance, societal norms, family expectations, and healthcare messaging—substantially influence women’s attitudes toward exercise. In some contexts, pregnancy is culturally framed as a period requiring rest and caution, which may discourage physical activity. Social support, partner attitudes, access to safe exercise environments, and trust in healthcare guidance also play pivotal roles. These factors may affect not only participation in exercise but also how women interpret and experience its psychological effects. Overall, while the awareness of the benefits of exercise in pregnancy is growing, there is a recognized *knowledge-action gap* where women may know that exercise is good but struggle to implement it due to cultural and social factors. In Croatia, prenatal healthcare is universally accessible through the public health system, and pregnant women routinely receive obstetric care. National guidelines recommending moderate physical activity during pregnancy are generally aligned with ACOG recommendations. Nevertheless, many pregnant women reduce their physical activity levels due to barriers such as fear of harming the fetus, insufficient tailored guidance, physical discomfort, fatigue, and persistent cultural beliefs that pregnancy requires reduced movement. Croatian research examining physical activity in pregnancy has primarily focused on obstetric outcomes, physical health indicators, and exercise prevalence, while the subjective psychological experiences associated with exercise remain underexplored. Similarly, although perinatal mental health is increasingly acknowledged within Croatian clinical practice, qualitative investigations capturing pregnant women lived emotional experiences are limited.

Existing evidence on exercise and perinatal mental health has been shaped by quantitative designs that emphasize measurable outcomes such as symptom reduction, prevalence rates, and psychological scale scores. Although, these studies demonstrate associations between prenatal exercise and mental health outcomes, they do not capture the subjective, contextual, and meaning-based dimensions of women’s experiences during pregnancy. Quantitative and mixed-methods approaches rarely illuminate how women perceive the psychological effects of exercise, how they integrate exercise into their coping strategies, or how it shapes their emotional and cognitive adaptation to pregnancy. In contrast, qualitative methodology enables an in-depth understanding of lived experiences, personal meanings, and psychological interpretations that cannot be fully captured through quantitative measures. The use of open-ended questions and probing allows participants to express themselves freely in their own words rather than selecting from predetermined options, as is typical in quantitative methods [17]. By capturing these subjective dimensions, qualitative study extends beyond symptom-based frameworks and contributes to a more nuanced understanding of the psychological significance of prenatal physical activity. The lack of experience-centered evidence restricts a comprehensive understanding of how exercise contributes to maternal psychological well-being. Qualitative research is uniquely suited to address these gaps by exploring lived experiences, personal interpretations, and psychosocial meanings. By focusing on women who engage in regular exercise, this study aims to provide deeper insights into the psychological dimensions of prenatal exercise. Understanding lived experiences may inform more person-centered prenatal counselling, mental health promotion strategies, and the development of supportive interventions in perinatal care. There are many studies in the literature on mental health during pregnancy, as well as on the importance of exercise during pregnancy. Although previous studies have demonstrated the positive effects of physical activity on mental health and well-being, little is known about pregnant women’s perceptions of the influence of exercise during pregnancy on their mental health. Therefore, the aim of this study is to explore pregnant women’s experiences and perceptions of the impact of exercise on mental health.

## 2. Materials and Methods

### 2.1. Study Design

A qualitative research design was used to explore pregnant women’s perceptions and experiences of the influence of exercise during pregnancy on their mental health. The research methodology was prepared in accordance with the Consolidated Standards for Reporting Qualitative Research [18] to enhance transparency and methodological rigor, particularly in describing the research team’s background, reflexivity considerations, participant recruitment, interview procedures, and the processes of data collection and analysis. The study was approved by the Ethics Committee of University North (approval number: 2137-0336-07-25-5; date: 4 December 2024), and all participants provided written informed consent.

### 2.2. Participants and Setting

A purposive sampling strategy was used to recruit pregnant women who met predefined eligibility criteria. Purposive sampling in qualitative research enables the selection of information-rich participants who can provide in-depth insights into the phenomenon under investigation—in this case, experiences of exercise during pregnancy and its perceived influence on mental health [19,20]. Participants were recruited from a clinical hospital setting during routine prenatal visits and were invited to participate voluntarily.

The study included pregnant women who attended regular prenatal visits at the Clinical hospital Sveti Duh, Zagreb, Croatia, between March and September 2025. Patients were informed about the ongoing study by the second author (MB) and were asked about their willingness to participate. Patients who agreed to participate provided their email address to the second author (MB), after which they were contacted by the first author (MF) via the same email address. The first author then informed the participants about the details of the study and sent them an informed consent form, which the participants signed and returned to the first author.

Inclusion criteria were pregnant women between the 30th and 37th weeks of gestation who engaged in regular exercise during pregnancy and were aged 18 years or older. Regular exercise was defined as at least 150 min of moderate-intensity physical activity per week, in accordance with the American College of Obstetricians and Gynecologists (ACOG) guidelines [21]. Exclusion criteria included pregnant women with medical or obstetric contraindications to exercise, as well as those with high-risk pregnancies requiring activity restriction. Women with conditions contraindicating exercise were excluded in accordance with ACOG recommendations [22].

### 2.3. Data Collection

Data were collected using semi-structured interviews, a method that allows for flexible and dynamic exploration by following up on interesting or unexpected responses, in contrast to more rigid structured interviews [23]. Each participant was informed that the interview would be conducted in an open format and would focus on her personal experiences and perceptions regarding the impact of physical activity on mental health. During the interviews, participants were encouraged to provide more detailed and reflective responses through the use of probing questions. All interviews were conducted by MF, and all participants gave permission for the interviews to be audio-recorded. The primary author conducted online interviews via Google Meet at times that best suited each participant. Each interview lasted approximately 30 min.

An interview guide with open-ended questions was developed based on existing literature related to exercise during pregnancy [3,13,15,24] and the authors’ clinical experience in the research area. The guide included the following questions:

1. Why do you exercise during pregnancy?
2. How do you feel during and after exercising during pregnancy?
3. What are your perceptions and experiences regarding exercise and its impact on mental health?
4. Is there anything related to exercise and mental health that we have not discussed and that you would like to add?

The questions were supplemented with prompts such as: “Can you tell me more about that?”, “What exactly do you mean?”, or “Could you explain that in more detail?”, with the aim of gaining a deeper understanding of the participants’ responses.

Pre-interviews with five participants allowed the researcher to refine interview skills, maintain focus on the research topic, and encourage respondents to share their genuine experiences and perceptions. These interviews were included in the analysis, as no modifications were made to the interview guide following the pre-interviews. After completing the fifth interview, MF and MB met to review the interviews and assess whether revisions to the interview guide were necessary. No new questions were added following this discussion.

The research team comprised investigators with professional backgrounds in physiotherapy and expertise in maternal health and exercise. Reflexivity and researcher positionality were actively addressed during all stages of the research. The authors discussed how their professional experiences and prior engagement with prenatal exercise research might shape the study design, interview process, and data interpretation. To enhance reflexive awareness and minimize interpretative bias, the research team engaged in regular analytic discussions, maintained reflexive notes, and revisited transcripts throughout coding and theme development. Interpretations were refined collaboratively to ensure that findings were grounded in participants’ accounts rather than researchers’ expectations.

### 2.4. Data Analysis

**Figure 1:**
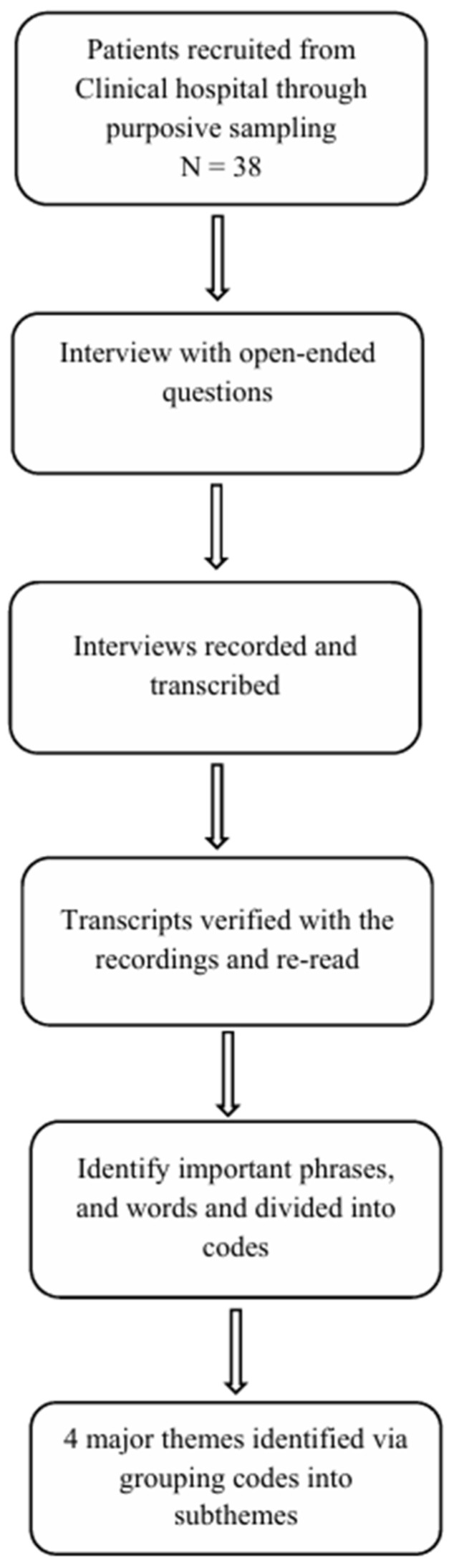
Flow chart of the study.

Data were analysed using an inductive thematic analysis approach following Braun and Clarke [23,25]. The selected analytical approach enabled an in-depth exploration of both manifest and latent similarities and differences within the data [23,25]. Although content analysis and thematic analysis are qualitative procedures, an inductive thematic analysis was selected because the study sought to identify and interpret patterns of meaning across participants’ lived experiences. Thematic analysis offers a deep, nuanced understanding of individuals’ experiences and realities regarding exercise in pregnancy and its influence on mental health, whereas content analysis provides a structured, focused examination of the content and its meanings. Furthermore, thematic analysis enabled exploration of latent psychological meanings, emotional interpretations, and experiential dimensions of exercise. The analysis comprised several phases: familiarization with the data, coding, identification of themes and subthemes, defining and reviewing themes and subthemes, and interpretation of their meaning in relation to the research question. The sample size for this study was determined using a saturation-based approach [26]. In accordance with established methods for assessing saturation in qualitative research, data collection was discontinued when no new themes or information emerged from subsequent interviews [26]. Following iterative interviewing and concurrent data analysis, data saturation was achieved, and recruitment was completed with a final sample of 38 pregnant women. Each audio-recorded interview was first listened to several times to gain an overall understanding of the data. The interviews were then transcribed verbatim and read repeatedly with the study aim in mind. Open coding was subsequently performed by writing notes and headings related to participants’ experiences with exercise directly into the text during the reading process. All codes were reviewed across the entire dataset and compared to identify similarities and differences. The next step involved data condensation and abstraction by grouping similar codes into subthemes, which were then organized and further abstracted into themes. The categorization process was continuously refined throughout the analysis to ensure the best possible fit with the data [23,25]. To ensure the stability of the data during the analysis process, the transcripts were reread multiple times [23]. The initial stages of analysis were conducted by the first author (MF). The second author (MB) read the interview transcripts and reviewed the codes and themes developed by the first author, with discrepancies discussed until consensus was reached. Finally, all authors discussed the themes and subthemes until full consensus was achieved. The study flow chart is presented in Figure 1, and examples of quotations, codes, themes, and subthemes are presented in the results section.

## 3. Results

Table 1 presents the sociodemographic characteristics of the participants. The women’s reported experiences and perceptions regarding the impact of exercise during pregnancy on mental health were classified into four main themes, and twelve subthemes were identified (Table 2). The four main themes are: Emotional Regulation and Mood Stabilization, Reduction of Anxiety and Depressive symptoms, Enhanced Self-Confidence and Body Acceptance and Increased Self-Efficacy and Sense of Control.

Analysis of the qualitative dataset revealed four interrelated themes that capture how pregnant women perceived the influence of exercise on their mental health. These themes illustrate the breadth of psychological benefits associated with maintaining exercise during pregnancy, including improved emotional stability, reduced anxiety and depressive symptoms, and enhanced feelings of confidence and capability.

Overall, the thematic analysis indicates that exercise during pregnancy plays a multifaceted role in promoting mental health. Across themes, participants described exercise as a source of emotional stability, confidence, and psychological resilience. Exercise was perceived not only as a physical practice but also as a powerful coping mechanism for managing anxiety, depressive symptoms, and the emotional demands of pregnancy.

### 3.1. Theme 1: Emotional Regulation and Mood Stabilization

Many participants described exercise as a key factor in stabilizing mood and helping them manage emotional fluctuations commonly experienced during pregnancy. Physical activities such as Pilates, aerobic exercise, and yoga often produced an immediate positive effect. Several women reported that exercise “lifted their mood” or helped them “start the day feeling mentally stronger”. For some, exercise also reduced irritability and prevented mood swings. Additionally, many participants emphasized that movement made difficult days more manageable and enhanced their sense of emotional uplift. Overall, participants characterized exercise as a natural strategy for maintaining emotional balance during a period of heightened vulnerability.

#### 3.1.1. Improved Mood

Regular exercise during pregnancy—particularly aerobic activities and Pilates—was perceived to have a positive influence on maternal mental health by improving mood both immediately after exercise sessions and gradually over time. Many women reported reductions in low mood, describing clearer thinking and emotional relief following physical activity. These effects may be related to reduced stress levels and improved emotional self-regulation. Furthermore, exercise was described as helping restore motivation and energy, fostering a more positive outlook during pregnancy.

*“The combination of movement and relaxation lifted my mood and reduced the heaviness I had been feeling.”*

(P4)

#### 3.1.2. Reduction in Mood Swings

Engagement in regular physical activity during pregnancy was associated with reduced mood swings, with women noting greater emotional stability even after short bouts of exercise. These brief sessions were often described as sufficient to regulate emotions and promote a sense of calm and balance.

*“When I started doing aerobic exercise every morning, I noticed my mood became much more stable. Before that, I felt overwhelmed and emotional. The routine helped me start the day calmly, and I felt mentally stronger and more balanced.”*

(P7)

#### 3.1.3. Increased Emotional Resilience

Exercise was also reported to contribute to increased emotional resilience, helping women cope more effectively with emotionally challenging days. Through improved stress management and emotional control, physical activity supported a stronger capacity to adapt to the psychological demands of pregnancy.

*“Regular exercise really helps me stay emotionally balanced and manage stress on tough days. It makes me feel strong and confident, not just physically, but also in handling my moods and the challenges of pregnancy.”*

(P11)

### 3.2. Theme 2: Reduction of Anxiety and Depressive Symptoms

Many women reported that exercise helped reduce nervousness, restlessness, and sleep disturbances on difficult days. Additionally, exercise facilitated better concentration at work and provided relief from negative thoughts and constant worry related to pregnancy, health, or labour. Women frequently noted that activities such as swimming and yoga helped “quiet the mind,” interrupting cycles of intrusive or spiralling thoughts. Exercise provided a mental break that allowed them to shift focus away from fears and uncertainties. Several women emphasized that movement helped them feel less overwhelmed and contributed to a calmer overall mental state. Some women described exercise as a valuable tool for coping with emotional heaviness during pregnancy. For these women, exercise served as an important source of relief from depressive feelings. Practices such as yoga offered moments of connection, motivation, and respite from emotional “fog”.

#### 3.2.1. Better Sleep Quality

Women described sleep as a key mediator through which exercise indirectly enhanced emotional stability and energy, while reducing irritability, fatigue, and symptoms of anxiety and depression. Regular exercise during pregnancy was linked to improved sleep quality, with participants reporting easier sleep onset and more restorative rest. Exercise appeared to relieve physical tension and mental restlessness, contributing to more consistent and refreshing sleep patterns.

*“Since I started exercising regularly, I fall asleep more easily and wake up feeling rested. Working out helps me release tension and relax my mind, so I have more energy and feel less irritable the next day. Better sleep has also made me less anxious and more emotionally balanced.”*

(P23)

#### 3.2.2. Relief from Intrusive Thoughts

Exercise provided mental relief from intrusive or repetitive thoughts, allowing women to experience periods of psychological calm and improved mental clarity. Focusing on movement and breathing helped shift attention away from distressing thoughts and supported emotional regulation.

*“When I swim, I can take a break from all the negative thoughts and just focus on my body and breathing. It helps me feel calmer and more in control of my emotions. After a session, my mind feels clearer, and I’m less weighed down by anxious or sad thoughts.”*

(P2)

#### 3.2.3. Reduced Pregnancy-Related Worry

Engaging in exercise helped reduce pregnancy-related worries by enhancing feelings of control, confidence, and reducing overthinking. This reduction in anxiety supported a more balanced emotional state and greater overall psychological well-being during pregnancy.

*“I was constantly overthinking- ‘Is the baby okay? Am I gaining too much weight?’ Exercise helped me break that cycle. After each workout, my mind felt more settled and less trapped in worried thoughts. That mental break was incredibly helpful.”*

(P33)

### 3.3. Theme 3: Enhanced Self-Confidence and Body Acceptance

Participants reported that exercise contributed to greater self-confidence, particularly as their bodies changed throughout pregnancy. Engaging in regular exercise helped women feel stronger and more comfortable in their bodies, counteracting feelings of insecurity. Completing a workout was often associated with a sense of achievement, which enhanced self-esteem. For some women, being active fostered a more positive perception of their physical capabilities and reduced concerns related to body image.

#### 3.3.1. Growing Confidence in a Changing Body

Participation in regular exercise during pregnancy supported growing confidence in a changing body, helping women develop greater acceptance and appreciation of physical changes. Through movement, women reported feeling more connected to their bodies, experiencing a better body image, and feeling reassured about their physical capabilities.

*“At the beginning of pregnancy, I felt insecure because everything about my body was changing so quickly. Joining a prenatal exercise class helped me regain confidence and made me feel connected to my body. Every session improves my overall body image during pregnancy.”*

(P9)

#### 3.3.2. Feeling Strong and Capable

Exercise enhanced women’s sense of personal strength and self-esteem during pregnancy. By fostering feelings of physical capability and body trust, physical activity reinforced positive self-perceptions and contributed to psychological empowerment. Feeling strong supported greater self-confidence and encouraged a more resilient and assured approach to the challenges of pregnancy and childbirth.

*“As I stayed consistent with prenatal Pilates, my confidence grew. I started seeing myself as someone who can adapt and stay strong throughout pregnancy. That shift in mindset reminded me that my body is strong and capable, and made me feel much better emotionally.”*

(P21)

#### 3.3.3. Increased Trust in Bodily Capabilities

Regular exercise during pregnancy strengthened women’s trust in their bodily capabilities, helping them feel more in control of physical changes and demands. This growing trust supported a sense of reassurance and confidence, reinforcing acceptance of the pregnant body and reducing fear related to physical limitations or childbirth.

*“Exercising regularly has helped me trust my body more and feel in control of the changes during pregnancy. It reassures me that I can handle the physical demands of pregnancy and childbirth. Being active also reduces my fear about limitations and helps me accept my pregnant body.”*

(P31)

### 3.4. Theme 4: Increased Self-Efficacy and Sense of Control

Many women expressed that staying active enhanced their sense of self-efficacy, particularly regarding their ability to manage pregnancy and prepare for labour. Establishing and maintaining an exercise routine provided structure, predictability, and a feeling of control during a period marked by constant physiological and emotional changes. Women described exercise as an activity they could “control,” helping them feel more capable, organized, and internally strong.

#### 3.4.1. Feeling Capable of Managing Pregnancy

Regular engagement in exercise enhanced women’s feelings of capability in managing pregnancy-related demands. Exercise supported a sense of competence in handling physical changes, emotional challenges, and daily activities, reinforcing self-efficacy and a greater sense of control throughout pregnancy.

*“Exercise boosted my belief in myself. I felt like, ‘Okay, if I can do this, I can manage the rest of pregnancy and even labour.’ It gave me a sense of capability I didn’t expect.”*

(P17)

#### 3.4.2. Confidence in Managing Pregnancy-Related Challenges

Exercise increased confidence in the ability to cope with common pregnancy-related challenges, such as fatigue, discomfort, and emotional fluctuations. This enhanced self-efficacy supported adaptive coping strategies and reduced feelings of helplessness.

*“Being active helped me feel more capable of handling fatigue, discomfort, and mood swings. It gave me confidence that I can cope with the daily challenges of pregnancy. Working out also helps me feel less helpless and more in control of my body and emotions.”*

(P1)

#### 3.4.3. Active Participation in Health and Pregnancy Outcomes

Participation in exercise fostered a sense of active involvement in one’s own health and pregnancy outcomes. By making intentional choices to remain physically active, women experienced greater empowerment and responsibility, strengthening their belief in their capacity to positively influence their pregnancy and preparation for childbirth.

*“I felt disconnected from myself during the first weeks of pregnancy. But when I started doing yoga, I felt like I reconnected with my body, which gave me a sense of control and responsibility over my pregnancy. That connection helped my mental health so much—it made me feel empowered and confident in preparing for childbirth and taking care of both myself and the baby at the same time.”*

(P29)

## 4. Discussion

The findings of this study highlight the significant and multidimensional influence of exercise on mental health during pregnancy. Across the dataset, participants described exercise as an accessible and effective strategy for managing emotional challenges, improving mood stability, and enhancing overall psychological well-being. These results are consistent with existing research suggesting that prenatal exercise contributes to reduced symptoms of depression and anxiety, improved emotional regulation, and enhanced self-perceptions during pregnancy [27,28,29].

Pregnancy can sometimes feel overwhelming and challenging for mothers, and this period is widely recognised as a time of heightened susceptibility to anxiety and depression [30,31,32,33]. The relationship between self-esteem, depression, and anxiety is well established across various populations, including pregnant women [30,34]. During pregnancy, women experience significant hormonal fluctuations that are essential for sustaining gestation but can also affect mood and emotional stability. These changes include imbalances in sex steroid hormones, as well as alterations in cortisol reactivity, both of which have been linked to mood swings, increased anxiety, and a higher risk of depressive symptoms [30,35]. Emotional well-being can also be influenced by the biopsychosocial environment, including stress related to anticipation of childbirth and shifts in relationship dynamics with partners, family, and friends [30,36]. Physical changes, such as weight gain and modifications in body image—particularly during the third trimester—may further contribute to negative body perception and reduced self-esteem [30,36,37]. The combination of hormonal, physical, and emotional changes increases susceptibility to comorbidities such as anxiety and depression during pregnancy [30].

### 4.1. Emotional Regulation and Mood Stabilization

One key finding of this study is the role of exercise in emotional regulation, particularly in stabilizing mood and preventing emotional fluctuations. Participants reported immediate mood-lifting effects after engaging in moderate-intensity exercise, which aligns with established evidence that exercise promotes the release of endorphins, reduces cortisol levels [38,39], and supports neurochemical processes associated with positive affect. While previous quantitative studies have demonstrated associations between exercise and improved mood during pregnancy, our findings provide qualitative depth, illustrating how women consciously recognize and utilize exercise as a strategy for managing emotional fluctuations. Participants did not describe mood benefits as abstract outcomes, but as tangible, day-to-day emotional shifts, such as “feeling mentally stronger” or “more emotionally stable”. This experiential dimension extends existing literature by revealing how pregnant women interpret exercise as an active form of emotional self-management rather than merely a health recommendation. Notably, activities such as Pilates or swimming were perceived as particularly beneficial, suggesting that pregnant women do not need to engage in high-intensity exercise to experience mental health improvements. The endorphin hypothesis proposes that exercise stimulates the release of endogenous opioid peptides in the brain, which help reduce pain and enhance mood. This neurochemical response may contribute to lower levels of worry and hopelessness. Recent research has demonstrated that endorphin release during exercise is associated with improved mood, supporting this hypothesis while also highlighting the need for further investigation to better understand the underlying mechanisms [27,40].

### 4.2. Reduction of Anxiety and Depressive Symptoms

The study also highlights the importance of exercise in reducing symptoms of anxiety, depression, and worry—three common psychological experiences during pregnancy. Many participants reported that exercise provided a cognitive distraction from intrusive thoughts and served as a mental reset, helping them manage uncertainties related to pregnancy and childbirth. These findings are consistent with literature emphasizing the anxiolytic effects of exercise, which may result from improved autonomic regulation, enhanced sleep quality, and increased feelings of control over bodily sensations [6]. Although the anxiolytic effects of exercise are well documented, this study highlights a novel insight: women perceived exercise not only as reducing symptoms, but as interrupting maladaptive thought patterns. The descriptions of “quieting the mind” and “breaking cycles of overthinking” suggest that exercise may play a role in perceived cognitive control during pregnancy. This observation extends existing evidence by emphasising the subjective psychological mechanisms through which physical activity supports mental well-being. Furthermore, Cole et al. (2025) reported that pregnant women who engaged in exercise experienced reduced anxiety, perceiving exercise as beneficial for both their own health and that of their fetus [13]. For pregnant women—who often face heightened medical, emotional, and social uncertainties—these psychological benefits may be particularly valuable. Stress reduction and improved sleep suggest that exercise supports mental health not only directly but also through indirect pathways. Improved sleep, which is often disrupted during pregnancy, was perceived to have secondary mental health benefits, including reduced irritability and anxiety. Similarly, reductions in physical tension contributed to psychological calm, supporting holistic models of mind–body interaction during pregnancy.

### 4.3. Enhanced Self-Confidence and Body Acceptance

Another finding of this study is the positive effect of physical activity on self-confidence and acceptance of bodily acceptance. Pregnancy involves profound physical changes including a rapid increase in body weight and body size, all occurring within a relatively brief period, that can challenge women’s body image [6,41,42]. These visible changes in appearance often negatively impact self-esteem, which is a known risk factor for both prenatal and postpartum depression [6,37]. Participants in the present study indicated that engaging in exercise helped them feel stronger, more capable, and more comfortable with their changing bodies. While body image disturbances during pregnancy are widely acknowledged, our findings suggest that exercise may facilitate positive embodiment, where women experience their changing bodies as functional and resilient rather than limiting. This represents a meaningful contribution, as participants framed exercise as strengthening not only physical fitness but also psychological reconciliation with bodily transformation. Such interpretations move beyond traditional biomedical discussions by positioning exercise as a mediator of identity and self-perception during pregnancy. This reinforces the notion that movement-based experiences can support positive embodiment during pregnancy and counteract feelings of physical vulnerability or loss of control. These findings are consistent with the study by Sun et al. (2018), which reported that women who engaged in physical activity during pregnancy experienced higher levels of body image satisfaction [43]. Engaging in physical activity appears to enhance psychological well-being through mechanisms such as improved mood, and increased perceptions of physical competence.

### 4.4. Increased Self-Efficacy and Sense of Control

The themes related to self-efficacy and perceived control further emphasize exercise as a mechanism through which pregnant women regain a sense of agency during a period characterized by unpredictability. Participants emphasized that maintaining an exercise routine generated feelings of competence, structure, and agency during a period often characterized by uncertainty and loss of control. Unlike prior studies that primarily measure self-efficacy quantitatively, this study captures how women subjectively construct a sense of capability through exercise. Women linked physical activity to confidence in managing pregnancy challenges and preparing for childbirth, suggesting that exercise may reinforce psychological resilience. This finding offers a novel conceptual contribution, highlighting exercise as a perceived tool for restoring autonomy and mastery rather than solely improving physical outcomes. Establishing a consistent exercise routine provided women with a sense of structure and accomplishment, which may buffer stress and enhance psychological resilience [30]. These findings suggest that exercise strengthens self-efficacy by offering opportunities for skill development and reinforcing feelings of competence—factors known to protect against perinatal stress, anxiety, and depression [6,32]. Participants described exercise as contributing to relief from anxiety and depressive symptoms, supporting evidence that exercise may improve mood by enhancing self-esteem, increasing energy, and providing opportunities for social engagement [6,37]. Many women viewed exercise as a valuable coping tool that made challenging days more manageable. This highlights the potential of exercise as a complementary strategy within broader perinatal mental health interventions. The beneficial effects of exercise on depressive symptoms may also be explained by biological mechanisms. Exercise elevates body temperature, which in turn increases brain temperature and promotes a sense of relaxation and calm. While the biological mechanisms underlying these effects are well described, they remain hypothesized rather than directly measured in this study. Additionally, exercise raises levels of β-endorphins, contributing to improved mood and overall emotional well-being [6,37]. Taken together, these findings illustrate that prenatal exercise offers a range of psychological benefits that extend beyond physical health. Participants perceived exercise as a stabilizing, empowering, and emotionally supportive practice, highlighting its potential as an important component of perinatal care. Healthcare providers should prescribe or encourage safe and structured prenatal exercise as a mental health strategy, integrating it into perinatal care plans and collaborating across disciplines, including obstetricians, midwives, physiotherapists, and mental health professionals. The results of this study demonstrate that exercise is not merely a form of physical activity but a holistic tool for enhancing psychological well-being and empowering pregnant women. Physically active women may differ from the broader pregnant population in important ways, including health behaviours, motivation, socioeconomic characteristics, and attitudes toward exercise. Although these differences introduce a degree of selection bias, the aim of this study was to capture rich experiential accounts of exercise related psychological processes among pregnant women who exercise regularly. Their narratives provide insight into how physical activity functions as a psychological resource during pregnancy, supporting emotional regulation, identity maintenance, and coping with physical and emotional changes. Nonetheless, this sample may not represent the full range of experiences across pregnancy, particularly among women who are less active or inactive. Future research should therefore explore the perspectives of these groups to achieve a more comprehensive understanding of exercise related psychological experiences during pregnancy. Also, future research could explore how contextual factors—such as social support, healthcare guidance, and accessibility of safe exercise programs—influence the mental health benefits reported here. Moreover, integrating qualitative insights with quantitative outcomes (e.g., measures of anxiety, depression, and self-efficacy) would offer a more complex understanding of the psychological mechanisms through which prenatal exercise exerts its effects. Future studies should also examine both the barriers and facilitators that influence women’s participation in physical activity during pregnancy, in order to inform more tailored and effective intervention strategies. Looking ahead, longitudinal designs could help to track changes in mental health outcomes across pregnancy and into the postpartum period, enabling the examination of temporal patterns and potential causal relationships. Healthcare providers should incorporate evidence-based recommendations on prenatal physical activity into routine antenatal consultations, ensuring that recommendations are individualized according to maternal health, pregnancy stage, and personal preferences. In addition, ongoing professional development should be offered to healthcare professionals to increase knowledge, confidence, and consistency in counselling pregnant women on safe and effective exercise practices. The participant sample in this study was relatively large and demonstrated diversity in terms of parity, age, and educational level and the interviews were conducted at the same time that participants were undergoing the experience, thereby reducing the likelihood of recall bias.

Nevertheless, the study has several limitations. First, the data are based on self-reported experiences; but this approach aligns with the aim of the study, which was to capture participants’ experiences in their own words. Second, the study does not account for socioeconomic, cultural, and environmental factors that influence access to safe and appropriate exercise resources. Variations in healthcare guidance, social support, and personal beliefs about exercise could shape experiences of pregnant women. Third, because the analysis is based on thematic interpretation rather than quantitative measures, it is not possible to determine causal relationships between exercise and mental health outcomes. The results illustrate perceived benefits rather than empirically verified effects.

The findings of this study have several implications for prenatal care, mental health promotion, and clinical practice. First, reframing exercise counselling: healthcare providers should present exercise not solely in terms of physical outcomes, but also as a strategy that supports emotional regulation, stress reduction, anxiety management and psychological well-being. Second, integration into mental health screening support. maternity services could incorporate exercise counselling into mental health screening contexts, recognizing physical activity as a complementary strategy rather merely a lifestyle recommendation. Participants’ descriptions of exercise as a *mental reset* and coping mechanism highlight its potential value in early supportive interventions. Third, interdisciplinary collaboration: coordination between obstetricians, midwives, physiotherapists, and mental health professionals may enhance professional support. Fourth, accessibility of structured programs: expanding availability of supervised prenatal exercise programs may improve both physical and psychological outcomes. Integrating exercise into perinatal care through clinical practice may contribute to more comprehensive, preventive, and person-centered maternal mental health strategies.

The transferability of the findings should be considered in light of the study’s sample characteristics. Participants were recruited from a single clinical setting and may represent women who were more engaged with healthcare services and potentially more motivated toward health-promoting behaviours. Therefore, the experiences and perceptions described may not fully reflect those of the broader pregnant population, particularly women with different socioeconomic, cultural, or health profiles.

The findings of our study indicate that exercise has a significant and multifaceted impact on mental health during pregnancy. These findings highlight the importance of healthcare professionals (e.g., nurses, midwives, and obstetricians) providing education and advice on prenatal physical activity, while taking careful account of each individual’s health status. Healthcare systems should facilitate the availability of supervised prenatal exercise programs in clinical and community settings. Additionally, tools such as educational resources, mobile applications, and follow-up consultations should be used to support adherence, address concerns, and monitor maternal well-being throughout pregnancy.

## 5. Conclusions

While the mental health benefits of prenatal exercise have been widely established through quantitative research, this study provides novel qualitative insight into how pregnant women experience, interpret, and assign meaning to these benefits. Rather than describing exercise merely as beneficial, participants articulated complex psychological functions, including emotional stabilization, cognitive relief from intrusive thoughts, enhanced body trust, and strengthened self-efficacy. These findings suggest that exercise is perceived not simply as a physical health behaviour but as a multidimensional psychological resource embedded within women’s daily coping strategies. By capturing women’s lived experiences, this study extends existing knowledge by illuminating the subjective psychological meaning of prenatal exercise. These findings underscore the importance of integrating exercise promotion into prenatal care not only from a physical health perspective but also as a preventive and supportive mental health strategy. Healthcare professionals should consider framing exercise recommendations in ways that acknowledge women’s emotional experiences, perceived barriers, and psychological motivations. Overall, recognizing these experiential dimensions may enhance the development of more person-centered, psychologically informed prenatal care strategies.

## Figures and Tables

**Table 1:** Sociodemographic data.

Sociodemographic Data	N = 38
Age	
20–24	3
25–29	11
30–34	18
35+	6
Parity	
Primiparous	20
Multiparous	18
Education	
High school	11
College	25
Doctorate	2

**Table 2:** Overview of Analyses and Examples.

Quotes	Codes	Subthemes	Themes
Why do you exercise during pregnancy? “After exercise, I feel happier and more energized. I notice fewer mood swings and feel stronger and more in control of my emotions after working out.”	Immediate mood lift after exercise Less irritability or emotional fluctuation Feeling stronger emotionally after exercise	Improved mood Reduction in mood swings Increased emotional resilience	Emotional Regulation and Mood Stabilization
How do you feel during and after exercising during pregnancy? “I sleep more deeply and wake up feeling refreshed.” “Exercise stops me from overthinking, and my mind feels clearer and calmer.”	More restorative or deeper sleep Mental relief from negative thoughts Reduced overthinking	Better sleep quality Relief from intrusive thoughts Reduced pregnancy-related worry	Reduction of Anxiety and Depressive Symptoms
What are your perceptions and experiences regarding exercise and its impact on mental health? “Through exercise, I have learned to accept my changing body. I feel empowered both physically and mentally, and I trust my body more to handle these changes.”	Acceptance of physical changes Psychological empowerment Confidence in handling pregnancy demands	Growing confidence in a changing body Feeling strong and capable Increased trust in bodily capabilities	Enhanced Self-Confidence and Body Acceptance
Is there anything related to exercise and mental health that we have not discussed and that you would like to add? “I can handle daily pregnancy challenges better. Exercise helps me feel less helpless and less fatigued. I feel confident that I can positively influence my pregnancy and delivery.”	Sense of control over pregnancy changes Coping with fatigue and discomfort Confidence in positively influencing childbirth preparation	Feeling capable of managing pregnancy Confidence in managing pregnancy-related challenges Active participation in health and pregnancy outcomes	Increased Self-Efficacy and Sense of Control

## Data Availability

The data presented in this study are available on request from the corresponding author. The data are not publicly available due to privacy or ethical restrictions.
